# Pembrolizumab-associated immune-related hepatitis: a retrospective case report analysis

**DOI:** 10.3389/fmed.2026.1823347

**Published:** 2026-06-17

**Authors:** Ke-qian Chen, Yang Chen, Jing-Fu Peng, Yi Huang, Xiang Liu

**Affiliations:** 1 Department of Clinical Pharmacy, Xiangtan Central Hospital, The Affiliated Hospital of Hunan University, Xiangtan, China; 2 Department of Clinical Pharmacy, The First People's Hospital of Loudi, Loudi, China; 3 College of Pharmacy, Shaoyang Polytechnic, Shaoyang, China

**Keywords:** immune checkpoint inhibitors, immune-related adverse events, immune-related hepatitis, PD-1 inhibitors, pembrolizumab

## Abstract

**Objective:**

Analyzing the clinical characteristics, potential mechanisms, and treatment strategies of pembrolizumab-induced immune-related hepatitis to improve clinical management and treatment safety.

**Methods:**

Case reports on pembrolizumab-induced immune-related hepatitis were retrieved from databases including PubMed, Science Direct, and Web of Science. Forty-two case reports were carefully reviewed and data on patient age, gender, primary disease, time to hepatitis onset, clinical manifestations, liver injury indicators, adverse reaction grading, treatment strategies, and prognosis were collected and analyzed.

**Results:**

Among these patients, 27 were male and 14 were female, and the average age was 64.2 years. The clinical manifestations included liver toxicity, neurological symptoms, skin reactions, systemic symptoms, and digestive symptoms. The time from initiation of pembrolizumab to the onset of immune-related hepatitis varied significantly. The shortest duration is 2 days, while the longest is 630 days. Corticosteroids were the primary treatment. The pathogenesis may be related to the dysregulation of liver immune tolerance.

**Conclusion:**

Before initiating pembrolizumab, clinicians should thoroughly evaluate patients' liver function and history of liver disease, and consider the hepatotoxicity of concurrent medications. During the use of pembrolizumab, regular monitoring of liver function should be conducted. If any abnormalities are found, appropriate treatment should be given promptly. Meanwhile, further research is needed to better understand pembrolizumab-induced immune-related hepatitis.

## Introduction

As a commonly used anti-cancer drug in clinical practice, immune checkpoint inhibitors (ICIs) exert anti-tumor effects by blocking immune checkpoint molecules and enhancing T cell activity (1). However, overactivation of T cells can cause immune-related adverse events (irAEs), which may lead to treatment interruptions or even patient death. IrAEs can affect multiple organ systems, including the liver, skin, nervous system, and endocrine system (2). Among them, immune-related hepatitis is one of the common irAEs. Common ICIs include Nivolumab, Pembrolizumab, Toripalimab, Sintilimab, Camrelizumab, Tislelizumab, Atezolizumab, and Durvalumab. Among them, pembrolizumab is the PD-1 inhibitor with the longest history and broadest application. In recent years, it has shown significant efficacy in treating melanoma, non-small cell lung cancer, esophageal cancer, and head and neck cancer (3). Compared to conventional chemotherapy, pembrolizumab offers higher selectivity and lower toxicity. With the widespread application of pembrolizumab, more and more cases have reported pembrolizumab-induced immune-related hepatitis, and even deaths have occurred. To a certain extent, severe immune-related hepatitis has hindered the clinical application of ICIs. Lack of awareness of this condition can seriously compromise patient health. Therefore, analyzing the clinical characteristics, occurrence mechanism, and treatment strategies for pembrolizumab-induced immune-related hepatitis is crucial for improving treatment safety. This article retrieved case reports on pembrolizumab-induced immune-related hepatitis from databases such as PubMed, Science Direct, and Web of Science. By systematically analyzing the patient characteristics, primary diseases, clinical manifestations, and adverse reactions, this article aims to provide references for clinical practice of pembrolizumab.

## Method

**Figure 1:**
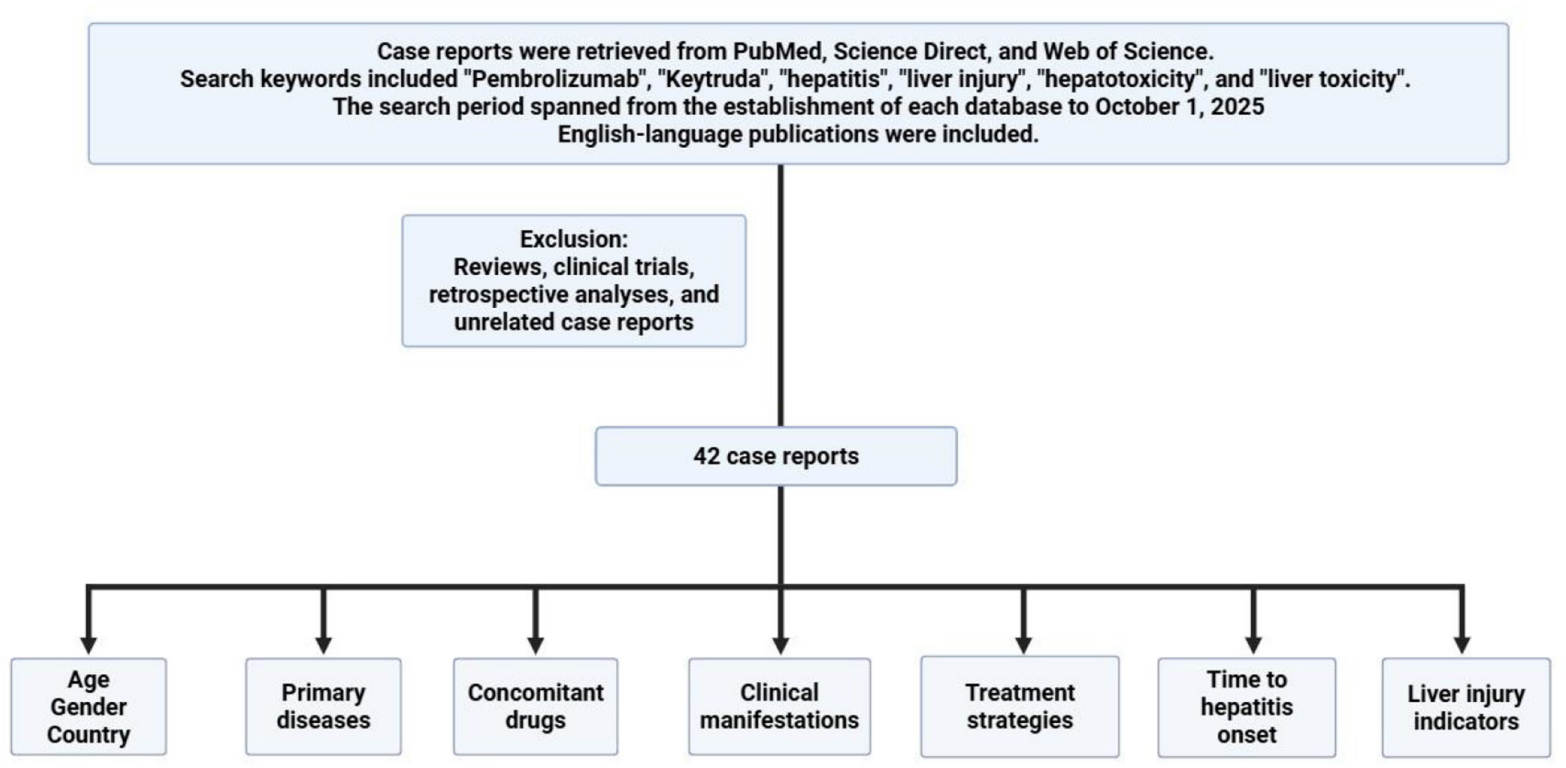
Flow chart of retrospective research.

Case reports on pembrolizumab-induced immune-related hepatitis were retrieved from databases including PubMed, Science Direct, and Web of Science from database inception to October 1, 2025. Only English-language publications were included. The following search strings were used with Boolean operators: PubMed: [“Pembrolizumab” (Title/Abstract) OR “Keytruda” (Title/Abstract)] AND [“hepatitis” (Mesh) OR “liver injury” (Title/Abstract) OR “hepatotoxicity” (Title/Abstract) OR “liver toxicity” (Title/Abstract)]. Web of Science: TS = (“Pembrolizumab” OR “Keytruda”) AND TS = (“hepatitis” OR “liver injury” OR “hepatotoxicity” OR “liver toxicity”). Science Direct: (“Pembrolizumab” OR “Keytruda”) AND (“hepatitis” OR “liver injury” OR “hepatotoxicity” OR “liver toxicity”). Exclusion criteria encompassed reviews, clinical trials, retrospective analyses, and unrelated case reports. Two independent researchers (KQC and YC) assessed the quality of included case reports using the Joanna Briggs Institute (JBI) critical appraisal checklist for case reports. Disagreements were resolved by a third reviewer (XL). All included case reports met ≥6 of 8 criteria (moderate to high quality). A retrospective research method was adopted (Figure 1). Forty-two case reports were carefully reviewed and data on patient age, gender, primary disease, time to hepatitis onset, clinical manifestations, liver injury indicators, adverse reaction grading, treatment strategies, and prognosis were collected and analyzed. This retrospective case report analysis was conducted following the PRISMA guidelines.

## Result

This article retrieved 42 case reports on pembrolizumab-induced immune-related hepatitis from databases such as PubMed, Science Direct, and Web of Science (4–45). We found 42 patients who developed immune-related hepatitis after treatment with pembrolizumab. The relevant information of these patients (age, gender, country, primary disease, time to hepatitis onset, clinical manifestations, liver damage indicators, adverse reaction grading, treatment methods) is as follows (Table 1): (1) Among the 42 patients, 27 were male and 14 were female (The gender and age of one patient were not reported). The youngest patient was 40 years old, the oldest was 86 years old, and the average age was

**Table 1:** Characteristics of patients with pembrolizumab-induced immune-related hepatitis.

Rank	Age/gender/country	Primary diseases	Clinical manifestations	Grade	Onset time	Liver injury indicators	Treatment strategies	Prognosis	Ref.
1	65/male/China	Lung cancer	Fever	4	2 weeks	ALT (2,912 U/L), AST (1,100 U/L)	Methylprednisolone, immunoglobulin	Recovery	(4)
2	48/female/Japan	Oral cancer	No date	4	2 days	ALT (972 U/L), AST (1,179 U/L)	No date	Recovery	(5)
3	69/male/Spain	Lung cancer	No date	No date	3 weeks	No date	Corticosteroids	Recovery	(6)
4	79/male/Japan	Lung cancer	No date	3	3 weeks	ALT (571 U/L), AST (549 U/L)	Prednisolone	Recovery	(7)
5	60/male/China	Liver cancer	No date	4	3 weeks	ALT (1,269 U/L), AST (1,193 U/L)	Hepatic protectants	Recovery	(8)
6	67/male/Belgium	Lung cancer	Dysarthria, hemiparesis	3	6 days	ALT (212 U/L), AST (223 U/L), ALP (97 U/L)	Methylprednisolone	Recovery	(9)
7	80/male/Japan	Renal cancer	Loss of appetite	3	7 months	ALT (535 U/L), AST (942 U/L), ALP (2,081 U/L)	Prednisolone	Recovery	(10)
8	42/male/Italy	Thymomas	Fever	3	20 days	ALT (258 U/L), AST (442 U/L)	Methylprednisolone	Recovery	(11)
9	86/male/China	Gastric cancer	Pruritus	3	10 days	No date	Methylprednisolone, ursodeoxycholic acid	Recovery	(12)
10	49/male/Japan	Renal cancer	No date	3	630 days	ALT (427 U/L), AST (443 U/L)	Prednisolone	Recovery	(13)
11	75/male/USA	Lung cancer	No date	3	28 days	ALT (518 U/L), AST (989 U/L)	Methylprednisolone, immunoglobulin	death	(14)
12	53/female/Sweden	Liver cancer	No date	4	6 months	ALT (940 U/L), AST (765 U/L), ALP (330 U/L)	Prednisolone, mycophenolate mofetil	Recovery	(15)
13	58/male/Japan	Oral cancer	Loss of appetite, abdominal pain	4	15 months	No date	Methylprednisolone, prednisolone, mycophenolate mofetil	Recovery	(16)
14	63/female/France	Melanoma	No date	2	3 weeks	ALT (136 U/L), AST (102 U/L), ALP (77 U/L)	Budesonide, ursodeoxycholic acid, corticosteroids	Recovery	(17)
15	68/male/China	Lung cancer	No date	3	13 days	ALT (233 U/L), AST (89 U/L), ALP (296 U/L)	Methylprednisolone, bicyclol	Recovery	(18)
16	40/male/Korea	Melanoma	No date	3	3 months	ALT (192 U/L), AST (107 U/L), ALP (225 U/L)	Methylprednisolone, prednisolone	Recovery	(19)
17	75/female/Spain	Lung cancer	Jaundice	4	3 months	ALT (1,692 U/L), AST (2,600 U/L), ALP (487 U/L)	Methylprednisolone	death	(20)
18	72/male/Japan	Lung cancer	No date	3	92 days	ALT (491 U/L), AST (233 U/L), ALP (778 U/L)	Methylprednisolone	Recovery	(21)
19	75/male/Spain	Lung cancer	Asthenia, myalgia, sweating, palpitations	4	3 weeks	No date	Dexamethasone, methimazole, propranolol	death	(22)
20	67/male/Australia	Melanoma	Fever	1	16 days	ALT (117 U/L), AST (69 U/L), ALP (2,275 U/L)	Methylprednisolone	death	(23)
21	74/male/Sweden	Colorectal cancer	Dry coughing	2	22 days	No date	Prednisolone	death	(24)
22	67/male/Portugal	Lung cancer	Jaundice, limb edema	4	2 days	ALT (3,557 U/L), AST (4,314 U/L), ALP (312 U/L)	Methylprednisolone	Recovery	(25)
23	74/male/China	Esophageal cancer	Blurred vision, blepharoptosis, Weakness	1	6 weeks	AST (94 U/L)	Methylprednisolone	Recovery	(26)
24	57/female/Italy	Renal cancer	Nausea, vomiting, mental confusion, sweating	4	17 days	ALT (935 U/L), AST (1,659 U/L)	Hydrocortisone	death	(27)
25	46/male/Japan	Lung cancer	No date	3	3 weeks	ALT (330 U/L), AST (179 U/L), ALP (133 U/L)	Prednisolone, ursodeoxycholic acid	Recovery	(28)
26	83/female/Japan	Renal/ureteral/bladder cancer	No date	3	1 mouth	ALT (422 U/L), AST (1,733 U/L), ALP (1,754 U/L)	Prednisolone	Recovery	(29)
27	73/male/USA	Head and neck cancer	Abdominal discomfort, diarrhea	3	6 weeks	ALT (205 U/L), AST (137 U/L), ALP (813 U/L)	Prednisone	Recovery	(30)
28	65/female/Japan	Lung cancer	No date	No date	2 mouths	No date	Prednisolone, azathioprine	Recovery	(31)
29	49/male/USA	Lung cancer	No date	3	4 mouths	ALT (508 U/L), AST (627 U/L), ALP (256 U/L)	Prednisone	Recovery	(32)
30	75/female/Taiwan (China)	Ureteral cancer	Bilateral ptosis	2	16 days	ALT (163 U/L), AST (258 U/L)	Methylprednisolone	Recovery	(33)
31	52/female/USA	Melanoma	No date	No date	15 weeks	No date	Prednisone	Recovery	(34)
32	47/female/Korea	Lung cancer	Left-sided ptosis, weakness	3	2 weeks	ALT (350 U/L), AST (524 U/L)	Prednisolone	Recovery	(35)
33	56/female/France	Lung cancer	No date	4	3 weeks	ALT (826 U/L), AST (650 U/L), ALP (623 U/L)	Prednisolone	Recovery	(36)
34	76/male/USA	Laryngeal cancer	No date	3	3 weeks	ALT (783 U/L), AST (413 U/L), ALP (444 U/L)	Prednisone, methylprednisolone, mycophenolate, thymoglobulin, ursodiol, antithymocyte globulin, tacrolimus	Recovery	(37)
35	50/male/Japan	Lung cancer	Jaundice	3	44 days	ALT (360 U/L), AST (283 U/L), ALP (862 U/L)	Methylprednisolone	death	(38)
36	69/male/Japan	Lung cancer	Fatigue	3	3 weeks	ALT (439 U/L), AST (779 U/L), ALP (591 U/L)	Ursodeoxycholic acid, methylprednisolone	Recovery	(38)
37	70/male/Japan	Lung cancer	No date	4	8 days	ALT (2,385 U/L), AST (3,803 U/L), ALP (652 U/L)	Steroids	Recovery	(40)
38	70/male/Australia	Melanoma	No date	4	2 mouths	ALT (1,242 U/L), AST (1,128 U/L), ALP (534 U/L)	Methylprednisolone, mycophenolate	Recovery	(41)
39	78/female/Australia	Breast cancer	Fatigue, anorexia, jaundice	4	12 mouths	ALT (1,519 U/L), AST (1,128 U/L), ALP (208 U/L)	Prednisolone	Recovery	(42)
40	57/female/Australia	Melanoma	Abdominal discomfort	3	6 mouths	No date	Methylprednisone, dexamethasone, antithymocyte globulin	Recovery	(43)
41	No date/No date/China	Liver cancer	Abdominal distension, loss of appetite	4	1 mouth	No date	Glucocorticoids, mycophenolate mofetil, artificial liver support	Recovery	(44)
42	52/female/Italy	Lung cancer	Diarrhea	No date	10 weeks	ALT (299 U/L), AST (147 U/L), ALP (161 U/L)	Prednisone	Recovery	(45)

64.2 years (Table 2). These descriptive findings indicate a higher proportion of older male patients in the reported cases. However, causal conclusions cannot be drawn due to the lack of denominator data (total patients treated with pembrolizumab) and potential reporting bias. (2) Forty-two patients were from Asia, Europe, North America, and Oceania, including Japan (12 cases), China (7 cases), the United States (5 cases), Australia (4 cases), Spain (3 cases), Italy (3 cases), France (2 cases), South Korea (2 cases), Sweden (2 cases), Belgium (1 case), and Portugal (1 case). This indicates a global occurrence, with a higher concentration of reported cases in Asia (Table 2). (3) Among the primary diseases, the most common cancers were lung cancer (19 cases), melanoma (6 cases), kidney cancer (4 cases), and liver cancer (3 cases). Additionally, other reported cancers included oral cancer (2 cases), ureteral cancer (2 cases), thymomas (1 case), gastric cancer (1 case), colorectal cancer (1 case), esophageal cancer (1 case), bladder cancer (1 case), head and neck cancer (1 case), laryngeal cancer (1 case), and breast cancer (1 case). These results suggest that pembrolizumab-induced immune-related hepatitis is prone to occur in various tumors (Table 3). (4) The time from initiation of pembrolizumab to the onset of immune-related hepatitis varied significantly. The shortest duration is 2 days, while the longest is 630 days. Notably, 59.5% of the patients occurred immune-related hepatitis within 4 weeks, but there were also some delayed cases of immune-related hepatitis, suggesting that long-term monitoring is necessary in clinical practice. (5) According to the Common Terminology Criteria for Adverse Events (CTCAE), pembrolizumab-induced immune-related hepatitis is classified as grades 1 to 4. In these case reports, grade 3 and grade 4 cases accounted for a relatively high proportion, suggesting that immune-related hepatitis was severe in most patients (Table 3). (6) Most patients showed abnormal liver function. The lowest level of ALT was 117 U/L, and the highest was 3,557 U/L. The lowest level of AST was 94 U/L, and the highest was 4,314 U/L (Table 3). (7) Corticosteroids (methylprednisolone, prednisone, dexamethasone) were the primary treatment. Additionally, other interventions included immunosuppressants (mycophenolate mofetil, azathioprine), hepatic protectants (Bicyclol), artificial liver support, immunoglobulins, and antithymocyte globulin (Table 4). (8) Among the 42 patients, 35 patients recovered after receiving active treatment, while 7 patients died (Table 4). The deceased patients often involved severe hepatitis and multiple organ failure, suggesting that severe immune-related hepatitis remains a significant risk in pembrolizumab therapy.

**Table 2:** General data of Patients with pembrolizumab-induced immune-related hepatitis.

Parameter	Classification	Value
Sex	Male	27 (64.3%)
	Female	14 (33.3%)
	No date	1 (2.4%)
Age	1–18 years	0 (0%)
	19–59 years	15 (35.7%)
	≥60 years	26 (61.9%)
	No date	1 (2.4%)
	Average age	64.2
Country	China	6 (14.3%)
	Japan	12 (28.6%)
	Spain	3 (7.1%)
	Belgium	1 (2.4%)
	Italy	3 (7.1%)
	USA	5 (11.9%)
	Sweden	2 (4.8%)
	France	2 (4.8%)
	Korea	2 (4.8%)
	Portugal	1 (2.4%)
	Taiwan (China)	1 (2.4%)
	Australia	4 (9.5%)
Region	Asia	22 (52.4%)
	Europe	11 (26.2%)
	Africa	0 (0%)
	America	5 (11.9%)
	Oceania	4 (9.5%)

**Table 3:** Clinical information of 42 included patients.

Parameter	Classification	Value
Primary diseases	Lung cancer	19 (45.2%)
	Liver cancer	3 (7.1%)
	Breast cancer	1 (2.4%)
	Gastric Cancer	1 (2.4%)
	Oral cancer	2 (4.8%)
	Renal cancer	4 (9.5%)
	Melanoma	6 (14.3%)
	Head and neck cancer	1 (2.4%)
	Ureteral cancer	2 (4.8%)
	Laryngeal cancer	1 (2.4%)
	Thymomas	1 (2.4%)
	Esophageal cancer	1 (2.4%)
	Bladder cancer	1 (2.4%)
**ALT**	≤ 40 U/L	0 (0%)
	41 U/L−100 U/L	0 (0%)
	101 U/L−1000 U/L	25 (59.5%)
	1001 U/L−2000 U/L	4 (9.5%)
	2001 U/L−3000 U/L	2 (4.8%)
	3001 U/L−4000 U/L	1 (2.4%)
	4001 U/L−5000 U/L	0 (0%)
	No date	10 (23.8%)
**AST**	≤ 40 U/L	0 (0%)
	41 U/L−100 U/L	3 (7.1%)
	101 U/L−1000 U/L	20 (47.6%)
	1001 U/L−2000 U/L	7 (16.7%)
	2001 U/L−3000 U/L	1 (2.4%)
	3001 U/L−4000 U/L	1 (2.4%)
	4001 U/L−5000 U/L	1 (2.4%)
	No date	9 (21.4%)
**Onset time**	<1 week	3 (7.1%)
	1–4 weeks	22 (52.4%)
	>4 weeks	17 (40.5%)
**Grade**	1	2 (4.8%)
	2	3 (7.1%)
	3	19 (45.2%)
	4	14 (33.3%)
	No date	4 (9.5%)

**Table 4:** Treatment and prognosis of 42 included patients.

Parameter	Classification	Value
Treatment strategies	Corticosteroids (methylprednisolone)	19 (45.2%)
	Corticosteroids (prednisolone)	13 (31%)
	Corticosteroids (dexamethasone)	2 (4.8%)
	Corticosteroids (no details)	1 (2.4%)
	Corticosteroids (prednisone)	5 (11.9%)
	Corticosteroids (hydrocortisone)	1 (2.4%)
	Mycophenolate mofetil	3 (7.1%)
	Mycophenolate	2 (4.8%)
	Ursodeoxycholic acid	4 (9.5%)
	Steroids	2 (4.8%)
	Immunoglobulin	2 (4.8%)
	Antithymocyte globulin	2 (4.8%)
	Hepatic protectants	1 (2.4%)
	Budesonide	1 (2.4%)
	Bicyclol	1 (2.4%)
	Methimazole	1 (2.4%)
	Propranolol	1 (2.4%)
	Azathioprine	1 (2.4%)
	Thymoglobulin	1 (2.4%)
	Ursodiol	1 (2.4%)
	Tacrolimus	1 (2.4%)
	Glucocorticoids	1 (2.4%)
	Artificial liver support	1 (2.4%)
	No date	1 (2.4%)
Outcome	Recovery	35 (83.3%)
	Death	7 (16.7%)

## Discussion

The pathological process of immune dysregulation in hepatic cells includes the impairment of self-antigen recognition, activation and attack of immune cells, and production of autoantibodies. The immune dysregulation of hepatic cells can also lead to an increase in multiple laboratory indicators through various mechanisms. For example, immune system attack leads to the disruption of the integrity of liver cell membranes, and ALT and AST are released into the blood, resulting in elevated transaminase (46). Excessive immune activity, with a large proliferation of B lymphocytes and secretion of immunoglobulins, leads to significant increases in globulin and IgG (47). At present, it is of great significance to distinguish these similar immune-related hepatitis. Autoimmune hepatitis is a chronic inflammatory disease. Its clinical features include elevated transaminase levels, positive autoantibodies, and lymphocyte infiltration in the liver (48). Drug-induced immune-related hepatitis does not mean that the drugs directly damage liver cells. Instead, certain drugs act as triggers, activating the abnormal immune system of the body, causing it to mistakenly attack its own liver cells, thereby triggering a liver inflammatory disease (49). The two diseases have similar clinical manifestations, but there are differences in their histochemistry. It is worth noting that pembrolizumab-induced immune-related hepatitis lacks the typical characteristics of autoimmune hepatitis (plasma cell infiltration, severe interface hepatitis, patchy necrosis, and rosette-like changes), and autoantibodies are usually negative (50).

The mechanism of pembrolizumab-induced immune-related hepatitis remains unclear and may be related to the dysregulation of hepatic immune tolerance. As a very tolerogenic organ, the liver contains multiple types of antigen presenting cells (APCs) that can present antigens and lead to immune tolerance (51). An increasing number of studies have also shown that these cells highly express PD-L1, which have the function of antigen presentation and can induce the production of Treg cell. For instance, plasmacytoid dendritic cell are abundant in the liver and they can upregulate PD-L1 in response to TLR and NLR agonists. By upregulating the expression of PD-L1, they were able to release IL-27 and promote the differentiation of Treg cell (52, 53). Antigen presentation is also a function of the kupffer cell (a liver-resident macrophage). Although kupffer cell can function as immunogenic APCs, antigen presentation by these cells is frequently accompanied by an upregulation of PD-L1, release of IL-10, TGF-β, prostaglandin E2, thereby inhibiting the activation of dendritic cell-mediated T cell and inducing the production of Treg cells (54). In addition to myeloid cells such as dendritic cell and kupffer cell, liver parenchymal cells are capable of antigen presentation. The tolerance-inducing properties of liver sinusoidal endothelial cells have been attributed to their ability to produce IL-10, TGF-β and PGE2 and to upregulate PD-L1 during antigen presentation to T cells (55).

In a word, these cells play an important role in regulating the body's tolerance to self-antigens and maintaining hepatic immune tolerance. PD-1 inhibitor (pembrolizumab) can block the PD-1/PD-L1 signaling pathway, leading to excessive activation of T cells and disrupting the physiological immune balance of the liver, thereby causing damage to liver tissues (56). In this case report series, older age, male gender, and lung cancer were frequently observed among reported cases. However, due to the absence of total treatment population data and the inherent limitations of case report analysis (reporting bias, lack of control group), these findings should be interpreted as descriptive observations rather than confirmed risk factors. Additionally, some other studies have also shown that genetic risk factors, liver function status before medication, underlying liver diseases, concomitant medication, and alcohol consumption are also risk factors for pembrolizumab-induced immune-related hepatitis.

Pembrolizumab-induced immune-related hepatitis can occur at any time during treatment. Its primary manifestation is often an asymptomatic elevation of AST and ALT levels, though some patients may present with elevated bilirubin and alkaline phosphatase (ALP) (12, 57). To characterize the pattern of liver injury, the R factor was calculated for each patient with available data. Among the 42 included patients, 22 patients (52.4%) provided the sufficient laboratory data (ALT and ALP) required for calculating the R factor. Based on the calculated R values, the distribution of liver injury patterns was as follows: hepatocellular pattern (R ≥5) in 12 patients (54.5%), mixed pattern (2 <R <5) in 4 patients (18.2%), and cholestatic pattern (R ≤ 2) in 6 patients (27.3%). These results indicate that pembrolizumab-induced immune-related hepatitis not only includes typical hepatitis, but also includes cholestatic and mixed patterns. More than half of the patients presented with the hepatocellular pattern, which is consistent with the pathological and physiological mechanism of PD-1 inhibitors activating T cells to directly attack liver cells. Unfortunately, due to the severe lack of data (nearly half of the data is missing), a large number of clinical studies will still be needed in the future to calculate the R factor. Generally speaking, pembrolizumab-induced immune-related hepatitis usually has no characteristic clinical manifestations, though non-specific symptoms like fever, fatigue, or loss of appetite may sometimes occur. Therefore, serum transaminase and bilirubin levels should be assessed before each treatment cycle. Additionally, other causes such as concomitant drugs (chemotherapy drugs), underlying diseases (viral hepatitis, fatty liver), and other factors (alcohol) should also be ruled out (58).

The primary treatment measure for pembrolizumab-induced immune-related hepatitis is to immediately discontinue the suspected drug, and then select the appropriate treatment drug based on the patient's condition. According to the guidelines of CTCAE and the National Comprehensive Cancer Network (Table 5) (59), for grade G3 or G4 liver damage, permanent discontinuation of pembrolizumab should be considered, and intravenous glucocorticoid therapy should be selected. When liver injury improves to grade 2, intravenous methylprednisolone succinate can be converted to oral prednisolone. After the condition improves, the dosage can be gradually reduced and discontinued over approximately 4 weeks.

**Table 5:** Grading of liver injury-related indicators.

Indicator	Grade 1	Grade 2	Grade 3	Grade 4
ALT	(1.5 3) × ULN	(3 5) × ULN	(5 20) × ULN	>20 × ULN
AST	(1.5 3) × ULN	(3 5) × ULN	(5 20) × ULN	>20 × ULN
AKP	(2 2.5) × ULN	(2.5 5) × ULN	(5 20) × ULN	>20 × ULN
GGT	(2 2.5) × ULN	(2.5 5) × ULN	(5 20) × ULN	>20 × ULN
TBIL	(1 1.5) × ULN	(1.5 3) × ULN	(3 10) × ULN	>10 × ULN

Several limitations of this study should be acknowledged. First, as a retrospective case report analysis, the findings are subject to publication bias (severe or unusual cases are more likely to be reported, while mild or asymptomatic cases may be underreported). Second, the absence of denominator data (total number of patients treated with pembrolizumab during the study period) prevents calculation of incidence rates or relative risks. Third, the small sample size (n=42) precludes multivariable analysis or generalizable conclusions. Fourth, only English language publications were included, potentially introducing language bias. Fifth, the lack of a control group (patients receiving pembrolizumab without hepatitis) means that all comparisons are purely descriptive.

## Conclusion

In conclusion, the clinical symptoms of pembrolizumab-induced immune-related hepatitis are not typical and difficult to diagnose. Although its incidence is relatively low, failure to intervene promptly can lead to fatal liver failure. Before initiating pembrolizumab, clinicians should thoroughly evaluate patients' liver function and history of liver disease, and consider the hepatotoxicity of concurrent medications. During the use of pembrolizumab, regular monitoring of liver function should be conducted. If any abnormalities are found, appropriate treatment should be given promptly. A limitation of this study is the small number of cases available for analysis. Further research is needed to better understand pembrolizumab-induced immune-related hepatitis.

## Data Availability

The original contributions presented in the study are included in the article/supplementary material, further inquiries can be directed to the corresponding authors.
